# Birth Experience, Postpartum PTSD and Depression before and during the Pandemic of COVID-19 in Russia

**DOI:** 10.3390/ijerph19010335

**Published:** 2021-12-29

**Authors:** Vera Yakupova, Anna Suarez, Anna Kharchenko

**Affiliations:** Faculty of Psychology, Lomonosov Moscow State University, 125009 Moscow, Russia; anna.suarez.fig@gmail.com (A.S.); an.an.Kharchenko@gmail.com (A.K.)

**Keywords:** postpartum depression, postpartum PTSD, obstetric violence, COVID-19 pandemic, birth experience

## Abstract

The aim of the study is to investigate the changes in the maternal healthcare system during the pandemic and their associations with maternal mental health in Russia. A sample of Russian women who gave birth during the first year of the COVID-19 pandemic (*n* = 1645) and matched controls, i.e., women who gave birth before the COVID-19 pandemic (*n* = 611), completed an anonymous Internet survey about recent childbirth. They were assessed for childbirth-related posttraumatic stress disorder (PTSD) and postpartum depression (PPD). Clinically relevant symptoms of PPD and PTSD were high before the pandemic and showed no significant change during the pandemic (*p* = 0.48 and *p* = 0.64, respectively). We found a notable increase in the frequency of obstetric violence (*p* = 0.015) during the pandemic, which, in turn, has a strong correlation with birth-related PTSD and PPD. The problem of ethical communication with patients among maternal healthcare professionals is acute in Russia, and it has been exacerbated by the pandemic. Family and doula support during labor can be a potential protective factor against obstetric violence.

## 1. Introduction

The pandemic has had a dramatic effect on the healthcare system around the globe, including maternal and childbirth practices. In 2020–2021 women have faced the threat of COVID-19 during pregnancy, labor, and after delivery. In January 2020, the Russian government introduced multiple anti-covid measures which varied across the regions [1]. These measures have significantly affected antenatal care and childbirth conditions. In February 2020, the hospitals across the country have restricted the possibility for the support person to attend birth, including the family members [2]. Furthermore, it was recommended to separate the infant and the mother right after delivery in case of the mother’s positive COVID-19 test [2]. These measures have resulted in a lack of support during and after labor and may have contributed to maternal perinatal mental health problems.

A growing body of research shows that mothers who gave birth during the pandemic had more clinically acute stress responses in comparison to those who had children in previous years [3,4,5]. In turn, higher levels of stress during birth were associated with more childbirth-related posttraumatic stress disorder (PTSD) symptoms and less bonding with the infant [3]. Among 1123 US perinatal women, participants reported clinically significant levels of depression (36.4%), generalized anxiety (22.7%), and PTSD (10.3%) during the pandemic [6]. In the Italian sample, 61.9% of pregnant women had high scores for depression after delivery during the pandemic [7]. 

In the qualitative study in Canada, women identified their hospital birth experience during the pandemic as important for their mental health [8]. Alterations in the maternal healthcare system have led to significantly reduced woman-centered care during the pandemic, opening avenues for discrimination of the COVID-19 positive women during labor [9]. Such discrimination experience, in turn, has been associated with higher levels of postpartum stress and PTSD [10]. The cross-national study from 64 countries, which included 6894 participants showed that women were primarily concerned about the restrictions for visitors after delivery (59%), the baby contracting COVID-19 (59%), lack of support during delivery (55%), and changes of the delivery plan due to COVID-19 (41%) [11].

The presence of a partner and a personal midwife or doula at birth has previously been associated with fewer medical interventions [12]. Higher numbers of medical interventions and obstetric violence, in turn, were shown to present a risk for developing postpartum PTSD and depression [13].

Childbirth-related PTSD may have long-term effects on maternal mental health [14]. Dikmen-Yildiz and colleagues have demonstrated that only 18.5% of women with birth-related PTSD showed recovery from the symptoms six months postpartum [15], while the other study demonstrated that parents were experiencing symptoms of posttraumatic stress as long as five years after birth [16]. There is evidence that postpartum PTSD can also affect mother-child attachment style [17] and infant development [18].

To our knowledge, this is the first study presenting data on maternal mental health and changes in the childbirth context due to the COVID-19 pandemic in the Russian Federation. We conducted the first study of the birth conditions, postpartum depression, and PTSD in Russia before the pandemic in January–February 2020. The following year we organized the follow-up study during the pandemic and investigated the changes in birth experience in Russia as well as their associations with birth-related PTSD and postpartum depression (PPD). While many circumstances that may affect maternal perinatal mental health are out of human control, such as pandemic and obstetrical complications, those related to proper support during labor and ethical considerations of the medical personnel may be modified. Therefore, it is important to study the possible risk factors for postpartum PTSD and depression and shed light on the situation in Russia. Thus, the aim of our research is to investigate the changes in birth experience, symptoms of postpartum PTSD and depression, and frequency of medical interventions and obstetric violence instances, due to the restrictive COVID-19 measures and COVID-19 disease diagnosis.

## 2. Materials and Methods

### 2.1. Procedure and Participants

The study included two stages: before the pandemic (January–February 2020) and during the pandemic (February–March 2021). During the period from January to February 2020 and February to March 2021, women received an invitation to participate in the study via thematic online and offline communities for expecting and new parents and childbirth education classes. The findings are based on the responses of a cross-sectional sample of 611 mothers of infants aged 0–13 months (M = 6.37) before the pandemic, and 1645 mothers of infants aged 0–14 months (M = 6.93) during the pandemic, who consented to participate in the study and completed the online survey. The participants were included in the study if they were 18 years old and over, were able to read and write in Russian, and had given birth within 14 months prior to the study.

### 2.2. Ethical Considerations

The study was conducted in accordance with the Declaration of Helsinki. Both study follow-ups were approved by the Ethical Committee of the Russian Psychological Society, Lomonosov Moscow State University. All women participated voluntarily and signed informed consent. The assessments were performed online via Google Forms tool.

### 2.3. Measures

#### 2.3.1. The Demographic, Pregnancy and Childbirth Experience Questionnaire

The survey included questions regarding the participants’ age at the time of testing, education level (primary, secondary, upper secondary/tertiary), place of residence and labor (Moscow and capital region/Other city in Russia with population > 1 million/Other city in Russia with population < 1 million/CIS country/Europe, USA or other), and marital status (married/cohabiting with partner/single). Respondents also answered questions regarding the pregnancy, such as parity, wellbeing during pregnancy, gestational age, time since birth, and delivery mode (vaginal/cesarean). 

Participants were asked to report the medical interventions during labor and their types.

1. episiotomy
2. amniotomy
3. use of synthetic oxytocin
4. epidural anesthesia

There was an option to add information about medical interventions. 

We asked the participants to mark the types of obstetric violence during the current childbirth, if they faced any. There was an option for the participants to add information about the obstetric violence experience.

1. medical interventions without patient’s consent and approval
2. verbal aggression and bullying
3. physical aggression (immobilization, forbiddance to drink)
4. threats and accusations
5. Kristeller maneuver
6. pain relief denial
7. ignoring the needs of the birthing woman

We also collected information about the presence of a support person during labor (none/partner/doula or private midwife/partner + doula or private midwife). 

Moreover, the follow-up survey also included questions about the COVID-19 diagnosis: whether any of the family members and/or the respondents themselves were diagnosed with COVID-19 during pregnancy, labor, or after delivery. The question about separation from the baby right after birth due to a positive COVID-19 test was also included.

#### 2.3.2. The City Birth Trauma Scale (CBTS)

We used the Russian version of the City Birth Trauma Scale (CBTS) [19] to assess birth-related PTSD symptoms according to DSM-5 criteria [20]. It is a self-report 31-item questionnaire, where 29 questions map onto DSM-5 diagnostic criteria and 2 questions relate to DSM-IV criteria. It includes 23 questions about the frequency of the PTSD symptoms scored on a Likert-type scale ranging from 0 (‘not at all’) to 3 (‘5 or more times’), covering four clusters of symptoms according to DSM-5: ‘Re-experiencing’ symptoms, ‘avoidance’ symptoms, ‘negative mood and cognitions’, and ‘hyperarousal’ symptoms. Additionally, two items assessed criterion A in accordance with DSM-5 (American Psychological Association [21] (APA), 2013) and another item assessed criterion A2 from DSM-IV [22] (American Psychiatric Association, 1994), scored as yes/no. Three questions assessed degree of distress, disability, and potential physical causes, scored as yes/no/maybe (sometimes), and two questions assessing onset (before childbirth/in the first 6 months following birth/later than 6 months after giving birth) and duration (less than 1 month, 1–3 months, more than 3 months) of symptoms. In the original study, the CBTS demonstrated high internal consistency (Cronbach’s α = 0.92); for the Russian version in the current study, Cronbach’s coefficient was α = 0.90. 

#### 2.3.3. Edinburgh Postnatal Depression Scale (EPDS) 

The EPDS [23] was developed to assess pre- and postnatal depressive symptoms. It is a 10-item questionnaire scale rated on a 4-point Likert scale, ranging from 0 to 3, which indicates how the mother has felt during the previous week. A score of 10 and higher is suggested to indicate clinically significant symptoms of depression [23]. In the present study, the Russian version [24] was used, with Cronbach’s α = 0.87.

#### 2.3.4. Covariates

We used maternal age at testing, level of education, family status, time after childbirth, gestational age, parity, and place of childbirth as covariates.

### 2.4. Statistical Analysis

Spearman’s correlation coefficient was used to estimate the relationship between postpartum PTSD and PPD symptoms as well as between these variables and the covariates listed above.

We explored the association between the PPD and postpartum PTSD symptoms and birth experience factors (medical interventions and obstetric violence) using generalized linear models. 

Multiple linear regression analysis examined the association between postpartum depressive and PTSD symptoms and the number of medical interventions and obstetric violence experiences. 

Pearson Chi-square tests were performed to compare the demographic and obstetric characteristics between the first and second follow-ups as well as to assess the associations between medical interventions and obstetric violence experience and the symptoms of postpartum depression and PTSD.

All analyses were performed using SPSS 25 software (IBM SPSS Statistics, Russian Federation).

## 3. Results

Demographic, obstetric, and childbirth characteristics for participants from the two cohorts are presented in Table 1. It shows that the samples did not differ in average age, level of education, and gestational age (*p*-values for all > 0.3). However, we found that participants who gave birth during the pandemic were more often married (Pearson Chi-square = 7.45, *p* = 0.024), more often gave birth in a specialized birth hospital under state insurance (Pearson Chi-square = 7.84, *p* = 0.02), more often gave birth for the first or second time (Pearson Chi-square = 7.62, *p* = 0.022), more often gave birth via cesarean (Pearson Chi-square = 6.80, *p* = 0.010), and, on average, longer time passed after the childbirth (Mean Difference = 0.56, *p* < 0.001). The majority of participants came from the capital Moscow region and big Russian cities (Table 1). 

As for the COVID-19 diagnosis, there were 111 (4.9%) participants who had the test-confirmed diagnosis during pregnancy, 35 (1.6%) participants who had it in the hospital during labor and the immediate postpartum period, and 121 (5.4%) women who reported contracting the infection after giving birth.

### 3.1. Medical Interventions and Obstetric Violence Experience

Table 1 further shows that the frequency of medical interventions during childbirth remained largely the same during the pandemic compared to the pre-pandemic levels (84.3% and 84.6%, respectively). With regard to types of medical interventions, there were no significant differences in the frequency of amniotomy, epidural anesthesia, and episiotomy (*p* > 0.10) (Table 1). However, there was a significant decrease by almost 5% in the use of synthetic oxytocin during the pandemic in comparison to pre-pandemic levels (Pearson Chi-square = 4.48, *p* = 0.034).

**Figure 1:**
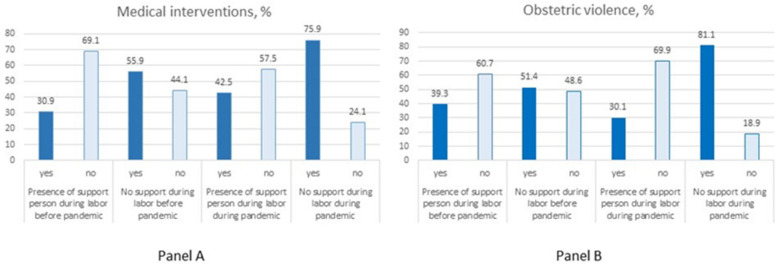
Frequency of medical interventions (panel A) and obstetric violence instances (panel B) depending on the presence/absence of a support person(s) during labor.

Figure 1, Panel A shows that having a support person present during labor was associated with decreased frequency of medical interventions both before (Pearson Chi-square = 5.73, *p* = 0.017) and during (Pearson Chi-square = 37.55, *p* < 0.001) the pandemic.

Table 1 also demonstrates that almost 5% of women experienced more instances of obstetric violence during the pandemic in comparison to the previous year (Pearson Chi-square = 6.06, *p* = 0.015). While the increase in the instances of medical interventions without consent, threats and accusations, denial of pain relief, and use of Kristeller maneuver did not reach statistical significance (*p* > 0.066), women reported significantly more experiences of verbal aggression and bullying when giving birth during the pandemic (Pearson Chi-square = 6.76, *p* = 0.009) and fewer instances of ignoring their needs during labor (Pearson Chi-square = 16.25, *p* < 0.001).

Figure 1, Panel B shows that partner and/or doula/private midwife support during labor was associated with decreased frequency of obstetric violence both before (Pearson Chi-square = 6.45, *p* = 0.014) and during (Pearson Chi-square = 20.77, *p* < 0.001) the pandemic.

### 3.2. Postpartum Depressive and PTSD Symptoms

There were no notable changes in the prevalence of clinically significant symptoms of either postpartum PTSD (17.5% vs 15.1%, Pearson Chi-square = 0.22, *p* = 0.64) or depression (43.9% vs 45.7%, Pearson Chi-square = 0.57, *p* = 0.48) during the pandemic in comparison to the pre-pandemic levels. PPD and postpartum PTSD symptoms were significantly correlated both before (Pearson correlation = 0.63, *p* < 0.001) and during pandemic (Pearson correlation = 0.62, *p* < 0.001).

**Figure 2:**
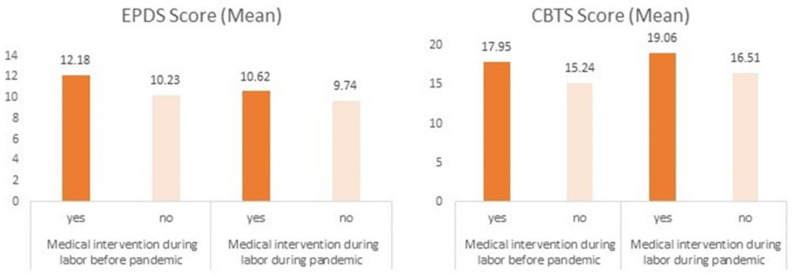
Association of postpartum depressive and PTSD symptoms and medical interventions during labor before and during pandemic. All values are adjusted for the maternal age at testing, level of education, family status, time after childbirth, gestational age, parity, and place of childbirth. EPDS stands for the Edinburgh Postnatal Depression Scale; CBTS stands for the City Birth Trauma Scale.

Figure 2 shows that symptoms of PPD and postpartum PTSD were higher among women with medical interventions during labor in both cohorts. The more interventions there were, the higher were PTSD symptoms both before (B = 1.02, 95% CI 0.20; 1.83, *p* = 0.014) and during pandemic (B = 1.03, 95% CI 0.55; 1.50, *p* < 0.001). Although to a lesser extent, there was also a positive correlation between PPD symptoms and the number of interventions both before (B = 0.68, 95% CI 0.25; 1.11, *p* = 0.002) and during pandemic (B = 0.41, 95% CI 0.16; 0.67, *p* = 0.002) (Figure 2).

**Figure 3:**
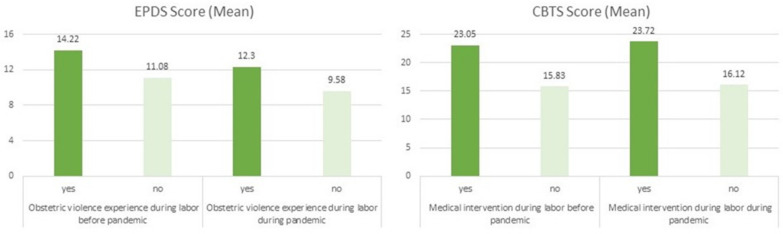
Association of postpartum depressive and PTSD symptoms and obstetric violence experience during labor before and during pandemic. All values are adjusted for the maternal age at testing, level of education, family status, time after childbirth, gestational age, parity, and place of childbirth. EPDS stands for the Edinburgh Postnatal Depression Scale; CBTS stands for the City Birth Trauma Scale.

Figure 3 further shows that both the PPD and PTSD symptoms were significantly higher among women who experienced obstetric violence during childbirth in both cohorts. The more instances of obstetric violence they experienced, the higher were the PTSD symptoms before (B = 5.09, 95% CI 3.81; 6.38, *p* < 0.001) and during the pandemic (B = 4.76, 95% CI 4.13; 5.39, *p* < 0.001). Similarly, symptoms of PPD increased the more instances of obstetric violence there were both in the first (B = 2.08, 95% CI 1.39; 2.78, *p* < 0.001) and second follow-up (B = 1.72, 95% CI 1.36; 2.07, *p* < 0.001).

There were no significant associations between having a support person present during labor and symptoms of postpartum PTSD and depression in either cohort (*p*-values for all > 0.19, data not shown). After adjustment for covariates, having a confirmed COVID-19 diagnosis was associated with more symptoms of PPD, if tested positive in the hospital during labor (F = 10.27, *p* = 0.001) or after delivery (F = 6.20, *p* = 0.013), but not during pregnancy (F = 0.96, *p* = 0.33). There were no associations between having the COVID-19 infection at any stage with symptoms of birth-related PTSD, nor were there significant associations with the COVID-19 status of family members at any stage (*p*-values for all > 0.092, data not shown).

## 4. Discussion

Our study was aimed to investigate the changes in birth experience due to the shifts in the maternity healthcare system during the pandemic and explore their associations with postpartum mental health. The main research variables were postpartum PTSD and depression, medical interventions, and obstetric violence during labor.

There is evidence of a higher prevalence of postpartum PTSD during the pandemic worldwide [3,6,25]. However, according to our results, there was no such trend in Russia, with less than a 2% increase in the rates of both birth-related PTSD and PPD. Yet it is important to note that the prevalence of 15–18% for postpartum PTSD and 43–46% for PPD in both of our cohorts is significantly higher than the one suggested in the recent meta-analysis, with a mean prevalence of 3.3% and 18.5%, respectively for high-risk groups [26]. It may suggest that women in Russia are at risk of developing mental health problems after giving birth in general, regardless of the changes due to the pandemic. 

Interestingly, the medical intervention rates did not change in light of the pandemic and remained at very high levels. Over 80% of women reported having at least one medical intervention during labor, with the total majority of births (>95%) taking place at the hospitals. Such high figures might reflect the highly medicalized birth culture in Russia which was not affected by the pandemic in either direction. Our results show significant associations between the number of medical interventions and postpartum depression and PTSD. A number of medical interventions can be necessary in case of birth complications, which, in turn, are related to posttraumatic stress [13]. However, some medical interventions may be routine procedures unnecessary for the birth progression, but rather driven by the hospital protocols and birth culture in Russia [27]. These procedures might also disrupt physical comfort, which is found to be an important protective factor for PTSD [28,29]. While labor in Russia is medicalized, there is an emerging trend to decrease the number of interventions during labor [30]. The lower frequency of synthetic oxytocin use during the pandemic may be the reflection of this trend and can be explained by more thorough birth preparation and women’s awareness of their legal rights [2].

The levels of obstetric violence before the pandemic in Russia were rather high with roughly every fourth woman experiencing at least one type of obstetric violence, according to our data. Sadly, the overall frequency of obstetric violence has further grown by more than 5% during the pandemic. Our research indicates a significant increase in bullying from the medical personnel, with 11.3% of women having faced bullying before the pandemic and 15.6% reporting such experience in the follow-up. One of the factors that may explain this increase is the COVID-19 related restrictions for the presence of support persons during labor. Our previous study showed that women with accompaniment experienced significantly less obstetric aggression in comparison to those giving birth without support [12]. Another possible factor is burnout of the medical professionals, which increased dramatically during the pandemic [31]. Obstetric violence, lack of ethical considerations, and poor communication remain the acute problem of the Russian maternal healthcare system [32]. Our data shows that obstetric violence is associated with higher PTSD and depression risks after delivery, which is consistent with other recent studies [33]. However, there is a lack of data on the topic across the countries. The reduction of obstetric violence rates is of key importance, as professional and careful communication of healthcare providers correlates with reduced rates of postpartum PTSD [34]. 

It is intriguing, however, that despite the overall increase in cases of obstetric violence experience, there were fewer instances of ignoring the needs of the women who were giving birth during the pandemic in comparison to the previous year. This result contradicts the worldwide data, where women report less care from medical staff during the pandemic [9]. We suggest that in line with the less frequent use of synthetic oxytocin, it may reflect the process of social changes in Russia, as women are preparing more thoroughly both for the labor process and communication with the medical staff [2]. They are more aware of their legal rights and demand appropriate healthcare. This is a very interesting trend that needs further research. 

One of the strongest effects of the COVID-19 related changes on birth experience is related to the possibility of the support person’s presence during labor. While in the Russian birth culture, the most common situation was hospital birth without any assistance of the partner or birth doula, there was an increasing trend of having a support person during labor [12]. Our study shows that before the pandemic almost 60% of women had at least one support person with them during labor, while less than 30% could have anyone present at their birth due to the COVID-19 restrictions. In the majority of hospitals in Russia, the partner either was not allowed to be present, or the conditions for his presence were not realistic. Sadly, the trend of support restrictions is unfolding worldwide and affects women’s mental health poorly [35]. It could be potentially risky because individual birth team availability is associated with reduced risk of postpartum PTSD [14] and lower rates of epidural anesthesia and cesarean births [36,37]. Our results are in line with this data, showing that the presence of a support person during labor was associated with lower obstetric violence and medical intervention rates.

Contracting COVID-19 infection has also affected maternal mental health directly. In our study testing positive for COVID-19 at the hospital or after delivery was associated with PPD, while it did not have such an effect during pregnancy, or when the infection was detected in a family member at any stage. We suggest that women, diagnosed with COVID-19 at maternity hospital before and after labor, could be anxious about separating from the infant right after birth, which may put them at risk for developing mental health problems. This result corresponds with the global data, where women name restriction for visitors after delivery (59%), the baby contracting COVID-19 (59%), lack of support during delivery (55%), and COVID-19 causing changes to the delivery plan (41%) as the main causes for anxiety [11]. Another important factor that may explain our results is the lack of mother-infant skin-to-skin contact immediately after delivery in case of detected COVID-19 infection. A number of studies show that skin-to-skin contact is associated with lower postpartum anxiety and depression, and traumatic stress symptoms [38,39,40]. Separation from the infant right after birth prevents such contact and may have long-term consequences for maternal mental health. 

The pandemic has remarkably changed the conditions of giving birth in Russia. There are significant differences in birth accompaniment and rates of obstetric violence. Our data shows that COVID-19 disease is not only a threat to maternal health and well-being per se, but it also presents a risk for poorer postpartum mental health due to restrictive measures which are introduced for the protection and prevention of the disease. Although there is a global discussion about the rates of violence against women during the pandemic, to date the evidence on the topic is scarce [41,42]. Our research provides essential data on the obstetric violence changes during the pandemic. 

Strengths of our study include considerable sample sizes of the two follow-ups, use of validated questionnaires, and thorough investigation of birth experiences. Furthermore, the inclusion of the questions regarding the COVID-19 diagnosis allows us to evaluate its prevalence and direct effect on maternal mental health in the follow-up. However, there are several limitations that should be taken into account when interpreting our results. First, we have collected data for both follow-ups exclusively online, with no direct contact of the researchers and the participants, which may have affected the women’s level of trust and, consequently, the reliability of their responses. Second, all the data is based on self-reports and lacks objective information on the participants’ health, including mental health and COVID-19 status, and obstetric history or medication use, which may limit their validity. Moreover, as we did not have access to the objective information on the clinical diagnosis of depression, we chose the commonly used EPDS score cut-off value of 10 or higher to estimate the prevalence of PPD in our samples. However, this cut-off point may be oversensitive and presents the risk of exaggerating the rate of PPD [43,44]. Therefore, further research using other diagnostic tools or more optimal EPDS cut-off for depression according to both DSM-5 and ICD-10 criteria is necessary. Finally, our samples were mainly represented by married women with higher education from big Russian cities which limits generalizability to different populations. Further studies including women with lower socioeconomic status from smaller cities are necessary to confirm the trends we have discovered in our samples. 

## 5. Conclusions

Overall, our study results show that the pandemic had a negative impact on birth experience in Russia. However, it remains unclear whether it was the effect of the COVID-19 infection itself, or rather of the restrictive measures which contributed to the already existing problem of maternal mental health in Russia. While there was no significant change in the rates of clinically relevant symptoms of postpartum depression and PTSD as they had been high already before the pandemic, we found a notable increase in the frequency of obstetric violence, which, in turn, has a strong correlation with birth-related PTSD and PPD. Our results indicate that the presence of support persons was associated with lower rates of obstetric violence and medical interventions. However, the restrictions due to the pandemic have limited the opportunity for having support during labor. 

The problem of ethical communication with patients among maternal healthcare professionals is acute in Russia, and it has been exacerbated by the pandemic. Family and doula support during labor can be a potentially protective factor against obstetric violence. Furthermore, the separation or threat of separation of mothers and their newborns due to the COVID-19 positive status of the mother should be re-evaluated as it may have a stronger long-term negative effect on their health than the infection itself. Therefore, the introduction of restrictive measures should take into account potential mental health risks for mothers and their children. 

## Figures and Tables

**Table 1:** Characteristics of the sample.

Characteristics		Women Gave Birth before Pandemic (*n* = 611)			Women Gave Birth during Pandemic (*n* = 1645)			*p*-Value
		Mean/N	SD/%	Range	Mean/N	SD/%	Range	
Age at testing (years)		31.17	4.54	18–45	30.98	4.42	19–50	0.39
Education	Upper Secondary/College	57	9.3%		135	8.2%		0.40
	Tertiary/University	554	90.7%		1510	91.8%		
Family Status	Married	559	91.5%		1547	94.0%		0.024
	Cohabiting with a Partner	33	5.4%		74	4.5%		
	Single	19	3.1%		24	1.5%		
Time After the Childbirth (Months)		6.37	3.42	0.2–12	6.93	3.30	0–14	<0.001
Gestational Age		39.47	1.67	28.0–43.0	39.40	2.04	0–43.0	0.45
Delivery Mode	Vaginal	472	77.4%		1183	71.9%		0.010
	Cesarean	138	22.6%		462	28.1%		
Place of Birth	Moscow and Capital Region	224	36.7%		403	24.6%		NA
	Other city in Russia with population > 1 million	262	43%		583	35.6%		
	Other city in Russia with population < 1 million				461	28.2%		
	CIS Countries	42	6.9%		98	6.0%		
	Europe/USA/Other	82	13.4%		91	5.6%		
Parity	1	359	58.8%		971	59%		0.022
	2	173	28.3%		522	31.8%		
	3+	79	12.9%		152	9.2%		
Type of childbirth plan	Birth in a specialized birth hospital under state insurance	344	56.3%		1020	62.0%		0.020
	Birth in a specialized birth hospital with a contract for a hospital or medical team of choice	250	40.9%		598	36.4%		
	Home birth	17	2.8%		27	1.6%		
Had at least one medical intervention during labor (yes)		517	84.6%		1386	84.3%		0.90
Number of medical interventions	1.62	1.19	0–5		1.57	1.19	0–7	0.40
Types of medical interventions	Amniotomy	279	45.7%		687	41.8%		0.10
	Epidural anaesthesia	244	39.9%		655	39.8%		0.96
	Use of synthetic oxytocin	230	37.6%		541	32.9%		0.036
	Episiotomy	116	19.0%		332	20.2%		0.55
Experienced at least one instance of obstetric violence during labor (yes)		138	22.6%		456	27.7%		0.015
Number of obstetric violence instances		0.32	0.68	0–4	0.42	0.82	0–4	0.007
Types of obstetric violence instances	Verbal aggression and bullying	69	11.3%		257	15.6%		0.009
	Medical interventions without consent	38	6.2%		137	8.3%		0.11
	Physical aggression (immobilization, forbiddance to drink)	NA	NA		19	1.2%		NA
	Threats and accusations	27	4.4%		104	6.3%		0.10
	Pain relief denial	19	3.1%		82	5.0%		0.066
	Use of Kristeller manoeuvre	19	3.1%		79	4.8%		0.082
	Ignoring the needs of the birthing woman	18	2.9%		15	0.9%		<0.001
Support person at labor (yes)		354	57.9%		443	27.0%		<0.001
Mode of birth support	No support	257	42.1%		1200	73.0%		<0.001
	Partner	217	35.5%		199	12.1%		
	Doula/Private midwife	74	12.1%		178	10.8%		
	Partner + doula/private midwife	63	10.3%		66	4.0%		
EPDS		9.88	6.07	0–26	9.46	6.13	0–30	0.15
CBTS		17.16	11.35	0–56	15.83	11.40	0–60	0.014
Confirmed COVID-19	During pregnancy	NA	NA		111	4.9%		NA
	During labor	NA	NA		35	1.6%		NA
	Postpartum	NA	NA		121	5.4%		NA

Note. *p*-values come from Pearson Chi-square (for nominal variables) and independent *t*-test (for continuous variables) statistics comparing the pre-pandemic (N = 611) and during pandemic (N = 1645) follow-ups. EPDS stands for the Edinburgh Postnatal Depression Scale; CBTS stands for the City Birth Trauma Scale.

## Data Availability

The data presented in the current study are available from the authors upon request.
